# Estimating the difference between structure-factor amplitudes using multivariate Bayesian inference

**DOI:** 10.1107/S2053273316003430

**Published:** 2016-03-30

**Authors:** Gergely Katona, Maria-José Garcia-Bonete, Ida V. Lundholm

**Affiliations:** a Department of Chemistry and Molecular Biology, University of Gothenburg, Gothenburg, 40530, Sweden

**Keywords:** experimental phasing, self-referencing, Bayesian model, time-resolved crystallography, Markov chain Monte Carlo algorithm

## Abstract

In experimental research referencing two or more measurements to one another is a powerful tool to reduce the effect of systematic errors between different sets of measurements. The interesting quantity is usually derived from two measurements on the same sample under different conditions. While an elaborate experimental design is essential for improving the estimate, the data analysis should also maximally exploit the covariance between the measurements. In X-ray crystallography the difference between structure-factor amplitudes carries important information to solve experimental phasing problems or to determine time-dependent structural changes in pump–probe experiments. Here a multivariate Bayesian method was used to analyse intensity measurement pairs to determine their underlying structure-factor amplitudes and their differences. The posterior distribution of the model parameter was approximated with a Markov chain Monte Carlo algorithm. The described merging method is shown to be especially advantageous when systematic and random errors result in recording negative intensity measurements.

## Abstract 2

A Bayesian model which uses a Markov chain Monte Carlo algorithm has been developed to estimate structure-factor amplitude differences.

## 1. Introduction   

There appears to be a strong dichotomy in the physical world between the observables and underlying wavefunctions (Dyson, 2007 ▸), and Bayesian models naturally lend an appropriate framework to discover the hidden parameters of the two-layered physical world. In X-ray crystallography the intensities of equivalent Bragg reflections are observed multiple times from which the directly unobservable structure-factor amplitudes can be estimated. Several X-ray crystallographic techniques exploit the fact that structure factors are dynamic and another (sub)structure can manifest itself as a difference in intensity observations. If the two sets of intensity observations are well separated in time or performed on different crystals there is a substantial risk that the systematic errors distort the difference amplitude estimates. To reduce the systematic errors between the observation sets, measurements can be taken from the same crystal and the intensities can be measured either simultaneously (González, 2003 ▸; Marinelli *et al.*, 2015 ▸) or in rapidly alternating cycles (Lundholm *et al.*, 2015 ▸; Westenhoff *et al.*, 2010 ▸).

For example, one can improve the measurement of anomalous differences through orienting the crystals such that the diffraction images contain a large number of symmetry-related Friedel pairs or through frequently re-orienting the crystals (inverse-beam geometry data collection strategy) (González, 2003 ▸).

In time-resolved pump–probe experiments, measurements without pump pulses or pumps with negative time delays are also frequently interspersed for referencing purposes (Schotte *et al.*, 2003 ▸; Srajer *et al.*, 1996 ▸). Difference intensities, unlike absolute intensity observations, are not immediately useful despite their frequently lower variance since the difference amplitudes cannot be determined without estimating the structure-factor amplitudes *F*_1_ and *F*_2_ [equation (1)]: 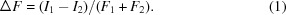
Since intensity observations *I*_1_ and *I*_2_ ultimately depend on structure-factor amplitudes *F*_1_ and *F*_2_ they can be incorporated in a single Bayesian model. Bayesian models are already applied to several different problems within crystallography. One of the first and probably most well known applications of Bayesian statistics was the treatment of negative intensities of Bragg reflections by French & Wilson (1978 ▸). The problem they addressed is that negative intensities may arise for weak reflections after subtracting the background even if a ‘true intensity’ can never be less than zero. Bayesian statistics are applied to this problem firstly to incorporate the positivity of the intensity into the prior distribution, and secondly to improve the estimates by assuming that the Wilson intensity distribution is usually (always) applicable (Wilson, 1949 ▸). French & Wilson’s method is already implemented in the program *cTRUNCATE* of the *CCP4* program suite (Winn *et al.*, 2011 ▸) and *XDSCONV* (Kabsch, 2010 ▸). Phasing methods also perform better by taking into account correlated errors in phasing experiments (Terwilliger, 1994 ▸; Terwilliger & Berendzen, 1997 ▸; Chiadmi *et al.*, 1993 ▸) and the Bayesian method was also introduced to (difference) refinement procedures (Terwilliger & Berendzen, 1996 ▸). Ursby *et al.* improved the estimates of difference amplitudes from poor data with a Bayesian methodology (Ursby & Bourgeois, 1997 ▸). Common to these approaches is that they consider structure amplitudes as observations with the exceptions of a few cases (Ursby & Bourgeois, 1997 ▸; French & Wilson, 1978 ▸; Chiadmi *et al.*, 1993 ▸). When intensity observations are the starting points, a common assumption is that there are no systematic errors in the measurements.

Moving from multiple intensity observations to structure-factor amplitudes using traditional merging approaches and following French & Wilson’s treatment involves significant loss of information, especially when intensity measurements are referenced to one other. The lost information is the covariance of the two measurements, which places important bounds on the possible values of *F*_1_, *F*_2_ and Δ*F*. These bounds are not used when the values of *F*_1_ and *F*_2_ are estimated from independent intensity sets *I*_1_ and *I*_2_ by standard procedures; therefore the accuracy gains of careful experimental design are partly lost. The covariance between *I*_1_ and *I*_2_ becomes especially useful when systematic or random errors force the observations to become negative. A univariate Bayesian treatment of strongly negative intensity observations results in a broad, nearly indistinguishable exponential posterior distribution with a mode/median/mean near zero. The potentially more sensitive Δ*F* estimate obtained from equation (1) is strongly affected by the small positive means/medians derived from the truncated univariate normal distribution, since this way *F*_1_ and *F*_2_ artificially inflate the Δ*F* estimates.

We propose a multivariate Bayesian model to treat pairwise intensity observations which we evaluate with a Markov chain Monte Carlo (MCMC) algorithm. The Bayesian algorithm performs the merging step of X-ray crystallographic data reduction and expects that the unmerged intensity observations are adjusted by the Lorentz–polarization and other correction factors and normalized to the same scale. The model even in its present form is relatively complex and yields a posterior distribution with multiple variables. The posterior probability is the integral over an *N*-dimensional space where *N* is the number of variables; this yields an exponentially increasing volume as a function of *N* according to the curse of dimensionality. This makes it impossible to search the space randomly to find the maximum. To be able to define the maximum of posterior space we used the MCMC algorithm to numerically estimate the multidimensional integral. Although a more rigorous Bayesian analysis would incorporate scaling factors and all unique reflections simultaneously, the computational costs are currently too high for all but the simplest crystal structures.

## 2. Results and discussion   

Two simulated intensity observation sets were generated based on the true value of structure-factor amplitudes *F*_1true_ and *F*_2true_.

Intensity observation sets 1 and 2 were defined as 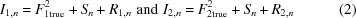
where *S_n_* consists of *n* random Gaussian variables with a mean μ_sys_ and σ_sys_ (sys = systematic), and sets *R*_1_ and *R*_2_ are generated from *n* random Gaussian variables with μ_1_ = μ_2_ = 0 and σ_ran_ (ran = random).

*S_n_* is equivalent to a collection of systematic errors affecting both observations whereas *R*_1,*n*_ and *R*_2,*n*_ represent the random errors that cannot be eliminated by referencing. It is most meaningful to use referencing as a data collection strategy if μ_sys_ ≠ 0 and/or σ_sys_ 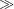 σ_ran_.

**Figure 1:**
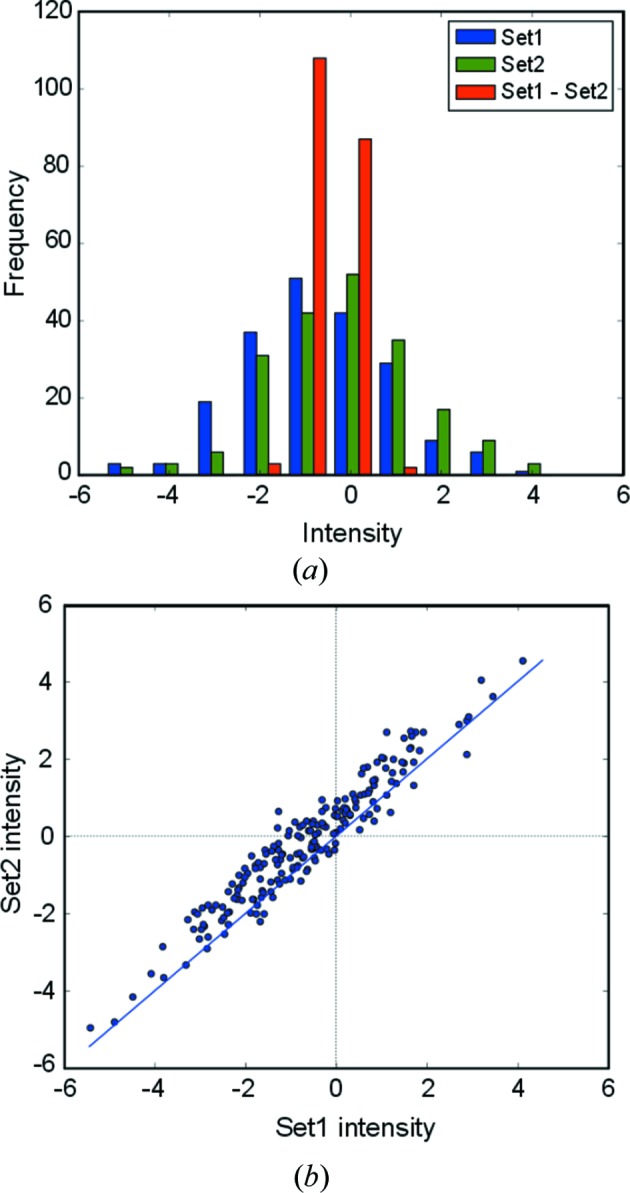
(*a*) Histogram showing the distribution of simulated data. Intensity observations of set 1 (blue) and set 2 (green) and their pairwise difference (set1 − set2) (red) are plotted with parameters *F*_1true_ = 0.1, *F*_2true_ = 0.7, μ_sys_ = −0.5, σ_sys_ = 1.5 and σ_ran_ = 0.3. (*b*) The same data set represented as a scatter plot. The observation pairs are shown as highly correlated blue dots parallel to the diagonal (blue line).

Fig. 1 ▸ illustrates the simulated intensity observations of two sets and the distribution of their pairwise differences. The off-diagonal displacement of data points carries specific information about the difference amplitudes and this displacement can even be utilized for pairs of negative intensity measurements.

We present an approach to improve the inference of structure-factor amplitudes given the systematic and random errors present in pairwise recorded intensity data. The basic idea is that we treat pairwise intensity observations as part of a bivariate joint normal distribution. The Bayesian model of these observations thus consists of the following stochastic and deterministic variables:


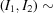
 MultivariateNormal 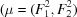,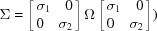 (Barnard *et al.*, 2000 ▸);


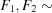
 Uniform 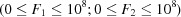;


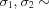
 logNormal 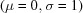 (Barnard *et al.*, 2000 ▸);


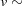
 Uniform 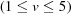;


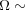
 LKJCorrelationMatrix(*v*) (Lewandowski *et al.*, 2009 ▸);


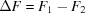
;where Ω is a family of positive definite correlation matrices. As *v* increases, the prior distribution of Ω increasingly concentrates around the unit correlation matrix. At ν = 1 Ω reduces to the identity distribution. σ_1_, σ_2_ represents the standard deviation of *I*_1_ and *I*_2_, respectively, and Σ is the covariance matrix of the multivariate normal distribution. Alternatively, the covariance matrix can be modelled directly with the stochastic Wishart distribution (Wishart, 1928 ▸), but using the current version of the PyMC3 library this led to numerical instabilities in the MCMC sampling. The added advantage of the model above is that prior distributions can be defined intuitively for σ_1_ and σ_2_.

**Figure 2:**
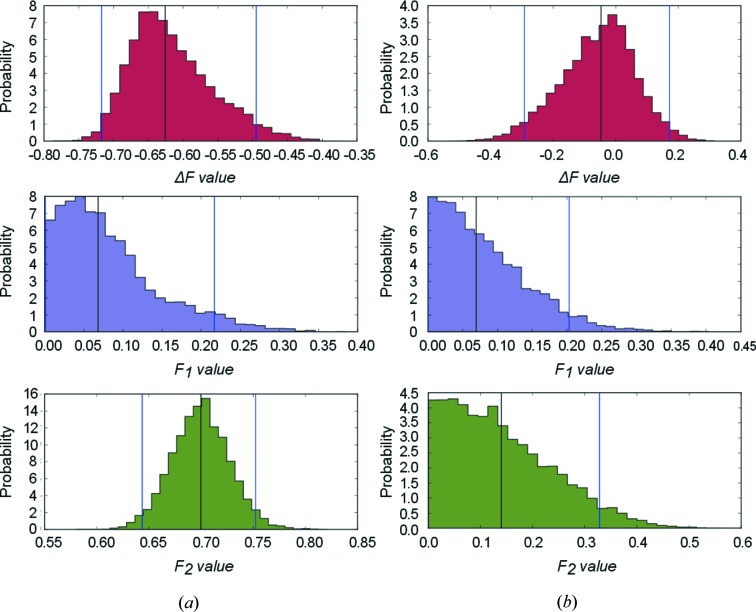
Posterior distributions of Δ*F*, *F*_1_ and *F*_2_ for the multivariate and univariate Bayesian models are shown in (*a*) and (*b*), respectively (first row in Table 1 ▸). Black lines indicate the median of the posterior distribution and blue lines show the borders of the credible 95% highest density interval (HDI), which does not necessarily exclude equal tails.

In the univariate Bayesian model, which is similar to the method developed by French & Wilson, the pairwise ordering of the data is ignored and intensity observations in *I*_1_ and *I*_2_ sets are modelled with univariate normal distributions:


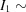
 Normal 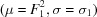;


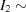
 Normal 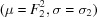;


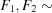
 Uniform (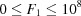; 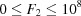);


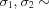
 logNormal 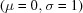;

In Table 1 ▸ each row represents data generated by specific parameters of equation (2). In total 200 referenced pairs were generated. We performed two MCMC simulations using the Metropolis stepping method consisting of 50 000 iterations from which the first 20 000 iterations were discarded (burn-in). No autocorrelation was detected in the remaining parameter chain. The results of a typical simulation are shown in Fig. 2 ▸.

Bayesian inference also allows prior information to be seamlessly incorporated into the models. The choice becomes especially important when the number of observations is low. In crystallography the intensity distribution is affected by the crystal symmetry and scattering angle, and appropriate prior distributions are determined from pools of similar reflections (French & Wilson, 1978 ▸).

**Figure 3:**
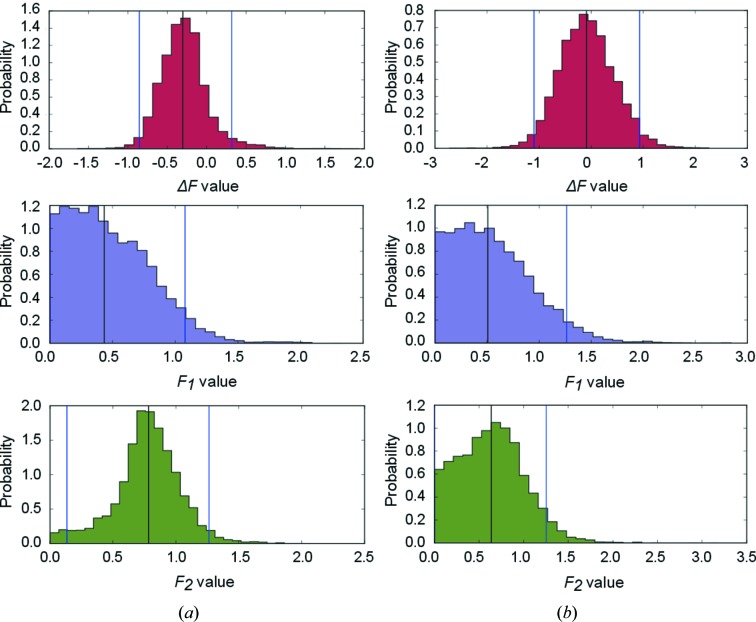
Posterior probability distributions of the variables after three observation pairs starting from a truncated prior normal distribution. The results from multivariate and univariate Bayesian models are shown in (*a*) and (*b*), respectively. Black lines indicate the median of the posterior distribution, and blue lines show the borders of the credible 95% highest density interval (HDI).

**Figure 4:**
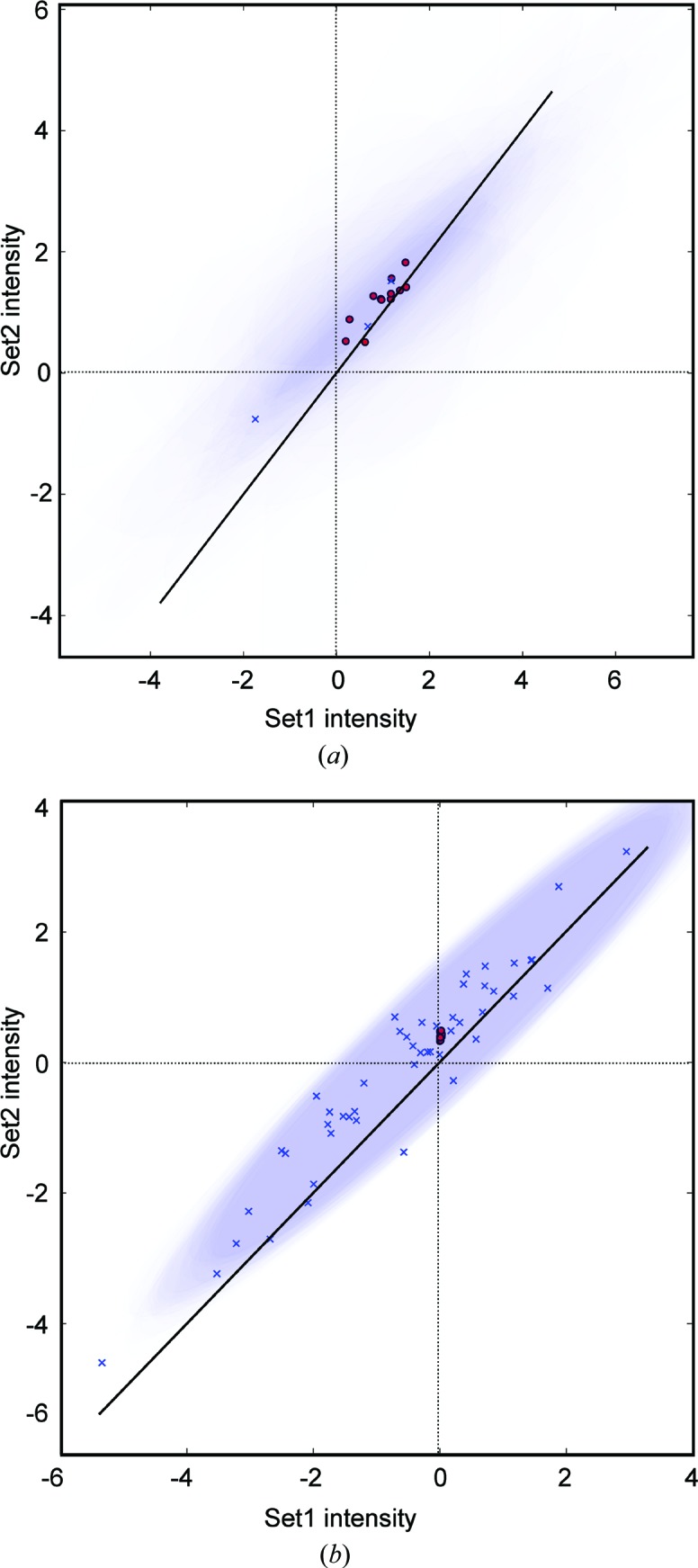
Posterior predictive check of the multivariate model using three (*a*) and 48 (*b*) observation pairs. Each joint posterior distribution is represented by a set of 95% isodensity ellipses (additive blue) from the MCMC trace. The coordinates of the centre correspond to the posterior samples of *F*_1_^2^ and *F*_2_^2^ (red circles). The length and direction of the ellipse axes are determined from the eigendecomposition (SciPy library function) of the covariance matrix of each MCMC trace sample. Blue crosses indicate the observations.

So far, we have used an unrealistically high number of observations for two reasons: firstly to minimize the influence of prior distribution and secondly to improve the reproducibility of the calculations from randomized data sets. As a next step we will compare two weakly informative prior distributions and a different number of observations. Table 2 ▸ and Fig. 3 ▸ illustrate what happens with the univariate and multivariate posterior distributions when the number of observations is reduced. The inference was then repeated with a uniform and weakly biased truncated normal prior distribution [*F*_1_ and *F*_2_ ∼ Normal (μ = 0.7; σ = 7 if *F*_1_ ≥ 0 and *F*_2_ ≥ 0)]. Already, at three observation pairs the multivariate model appears to provide a better difference amplitude estimate than the univariate model and the estimates improve with the increasing number of observations (Fig. 4 ▸). The univariate model becomes worse as it is updated with more observations and even a (weakly) biased prior distribution cannot prevent this deterioration. Table 2 ▸ also illustrates the importance of multiplicity and partly reinforces the commonly held view that a high number of observations improves the accuracy and precision of intensity/structure-factor amplitude estimates (Diederichs & Karplus, 2013 ▸). It also shows that in certain cases this is only true for the multivariate model. The main explanation for this counter-intuitive behaviour is the presence of negative observations in the data sets and the multivariate model clearly offers a better treatment for these.

A significant drawback of the MCMC algorithms is that they are computationally demanding. We used the PyMC3 Python library (Patil *et al.*, 2010 ▸) to perform 50 000 MCMC (Metropolis stepping) iterations on a Linux workstation (i7-3970X CPU at 3.50 GHz clock frequency) which took 35 s and 13 s for the multivariate and univariate model, respectively. The Theano dependency (Bergstra *et al.*, 2010 ▸) of the PyMC3 library allows for efficient evaluation of mathematical expressions involving multidimensional arrays as well as the use of graphical processing units (GPU) if available, but in this work the GPU was not used. The calculation for each reflection can be independent and can therefore be easily parallelized. Merging a crystal structure with 10 000 unique reflections takes approximately 8 h with 12 parallel processes and the above-mentioned CPU. Fortunately there is an ever-increasing family of related Bayesian algorithms that promise more efficient sampling/approximation of the posterior distributions, for example the No-U-Turn Sampler (NUTS) (Hoffman & Gelman, 2014 ▸) MCMC algorithm, Variational Bayes (Attias, 1999 ▸) and the Laplace approximation (Azevedo-Filho & Shachter, 1994 ▸).

For example, with the NUTS sampling method the length of MCMC traces can be reduced by an order of magnitude while achieving similarly accurate posterior estimates as with the Metropolis sampling. The current implementation of NUTS in PyMC3 frequently suffered from numerical instabilities in our hands, but this is likely to change as the library emerges from the beta development phase. Better difference amplitude estimates can mean the difference between a crystal structure solved by MAD/SAD (multiwavelength anomalous dispersion/single-wavelength anomalous dispersion) phasing and failure. Often it is much more time consuming to obtain better diffraction data and therefore the computational costs should be seen from that perspective.

Referencing is the basic principle behind the inverse-beam data collection strategy where pairs of reflections connected through Friedel’s symmetry are recorded in short succession. This way the difference in X-ray radiation damage between the intensity measurement pairs is minimal and the two measurements contain more information about the anomalous structure-factor difference. Their intensity estimates can be dramatically improved by taking into account the covariate of the weak reflection. Similarly, in time-resolved pump–probe studies the most detailed structural information regarding atomic displacements is contained in weak reflections at high resolution and these are strongly affected by Bayesian assumptions during data processing. These experiments are very often performed on the same sample in short succession and it is therefore straightforward to incorporate the multivariate Bayesian treatment presented in this paper.

## 3. Conclusions   

We have shown that a multivariate Bayesian model can provide more accurate structure-factor amplitude estimates from pairwise recorded diffraction intensities than univariate modelling. We also demonstrated that this multivariate model can be efficiently evaluated by an MCMC algorithm. We anticipate that the accuracy gains will lead to improved phasing results and more detailed difference electron-density maps in time-resolved pump–probe diffraction experiments.

## Figures and Tables

**Table 1:** Relation of Bayesian estimators (median of the posterior distribution) to their true value determined under different error models The width of their credible interval is shown in parentheses (HDI, highest density interval, 95%). Bold values indicate a closer match to the target value.

*F*_1true_	*F*_2true_	μ_sys_	σ_sys_	σ_ran_	*F*_1_,_multivariate_	*F*_1,univariate_	*F*_2,multivariate_	*F*_2,univariate_	Δ*F*_multivariate_	Δ*F*_univariate_
0.1	0.7	−0.5	1.5	0.3	0.07 (0.26)	**0.09** (0.24)	**0.71** (0.10)	0.16 (0.38)	**−0.63** (0.25)	−0.08 (0.51)
0.2	0.5	−1.0	1.5	0.3	0.06 (0.17)	0.06 (0.18)	**0.48** (0.16)	0.07 (0.20)	**−0.41** (0.22)	−0.00 (0.33)
1.0	0.1	0.0	2.0	1.0	**0.98** (0.19)	0.85 (0.37)	0.16 (0.38)	**0.14** (0.38)	**0.82** (0.38)	0.70 (0.57)
3.0	0.5	−5.0	6.0	3.0	**2.84** (0.25)	1.96 (0.49)	0.09 (0.32)	**0.13** (0.37)	**2.74** (0.45)	1.81 (0.64)
0.6	0.1	−0.5	1.2	0.4	**0.63** (0.13)	0.09 (0.26)	0.06 (0.17)	0.06 (0.18)	**0.56** (0.21)	0.03 (0.37)

**Table 2:** The influence of the number of observations and the choice of prior distribution on the posterior distribution of the parameters The data were generated with the following parameters: *F*_1true_ = 0.1, *F*_2true_ = 0.7, μ_sys_ = −0.5, σ_sys_ = 1.5 and σ_ran_ = 0.3 (Fig. 1 ▸). The width of their credible interval is shown in parentheses (HDI 95%). Bold values indicate a closer match to the target value.

	No. observations	*F*_1,multivariate_	F_1,univariate_	*F*_2,multivariate_	*F*_2,univariate_	Δ*F*_multivariate_	Δ*F*_univariate_
Uniform prior (0–10^8^)	3	**0.43** (1.10)	0.50 (1.26)	0.78 (1.09)	**0.64** (1.26)	**−0.31** (1.10)	−0.10 (2.09)
	6	**0.30** (0.82)	0.34 (0.90)	**0.79** (0.68)	0.45 (0.99)	**−0.45** (0.67)	−0.07 (1.58)
	12	**0.30** (0.71)	0.33 (0.79)	**0.77** (0.44)	0.57 (0.97)	**−0.45** (0.53)	−0.20 (1.36)
	24	**0.20** (0.57)	0.23 (0.61)	**0.64** (0.42)	0.30 (0.72)	**−0.42** (0.47)	−0.06 (1.11)
	48	**0.13** (0.38)	0.14 (0.41)	**0.66** (0.26)	0.21 (0.53)	**−0.51** (0.37)	−0.05 (0.81)
							
Truncated normal prior for *F*_1_ and *F*_2_, μ = 0.7, σ = 7.0	3	**0.43** (1.08)	0.51 (1.26)	0.78 (1.13)	**0.63** (1.25)	**−0.30** (1.17)	−0.09 (2.03)
	6	**0.31** (0.87)	0.33 (0.91)	**0.79** (0.72)	0.44 (0.99)	**−0.45** (0.69)	−0.08 (1.58)
	12	**0.29** (0.74)	0.32 (0.79)	**0.77** (0.45)	0.57 (0.97)	**−0.46** (0.56)	−0.21 (1.39)
	24	**0.20** (0.57)	0.23 (0.62)	**0.64** (0.41)	0.30 (0.72)	**−0.42** (0.45)	−0.05 (1.13)
	48	**0.14** (0.40)	0.15 (0.41)	**0.67** (0.27)	0.22 (0.54)	**−0.50** (0.39)	−0.06 (0.80)
